# DNA methylome and transcriptome profiling reveal key electrophysiology and immune dysregulation in hypertrophic cardiomyopathy

**DOI:** 10.1080/15592294.2023.2195307

**Published:** 2023-04-02

**Authors:** Xiaoyan Li, Hailang Fan, Xiantao Song, Bangrong Song, Wenxian Liu, Ran Dong, Haikun Zhang, Shicheng Guo, Hao Liang, Steven J. Schrodi, Xuebin Fu, Sunjay Kaushal, Yanlong Ren, Dake Zhang

**Affiliations:** a Beijing Anzhen Hospital, Beijing Institute of Heart, Lung and Blood Vessel Diseases, Capital Medical University, Beijing, China; b Key Laboratory of Biomechanics and Mechanobiology, Ministry of Education, Beijing Advanced Innovation Center for Biomedical Engineering, School of Engineering Medicine, Beihang University, Beijing, China; c Department of Cardiology, Beijing Anzhen Hospital, Capital Medical University, Beijing, China; d Beijing Lab for Cardiovascular Precision Medicine, Beijing Anzhen Hospital, Capital Medical University, Beijing, China; e Department of Cardiac Surgery, Beijing Anzhen Hospital, Capital Medical University, Beijing, China; f Department of Medical Genetics, University of Wisconsin-Madison, Madison, WI, USA; g Computation and Informatics in Biology and Medicine, University of Wisconsin-Madison, Madison, WI, USA; h Department of Pediatrics, Ann and Robert H. Lurie Children’s Hospital, Chicago, IL, USA; i Department of Cardiovascular-Thoracic Surgery, Feinberg School of Medicine, Northwestern University, Chicago, IL, USA

**Keywords:** Cardiac electrophysiology, DNA methylation, hypertrophic cardiomyopathy, immune responses, myocardial remodelling, transcriptome

## Abstract

Hypertrophic cardiomyopathy (HCM) is the most common inherited heart disease. However, a detailed DNA methylation (DNAme) landscape has not yet been elucidated. Our study combined DNAme and transcriptome profiles for HCM myocardium and identify aberrant DNAme associated with altered myocardial function in HCM. The transcription of methylation-related genes did not significantly differ between HCM and normal myocardium. Nevertheless, the former had an altered DNAme profile compared with the latter. The hypermethylated and hypomethylated sites in HCM tissues had chromosomal distributions and functional enrichment of correlated genes differing from those of their normal tissue counterparts. The GO analysis of network underlying the genes correlated with DNAme alteration and differentially expressed genes (DEGs) shows functional clusters centred on immune cell function and muscle system processes. In KEGG analysis, only the calcium signalling pathway was enriched either by the genes correlated with changes in DNAme or DEGs. The protein-protein interactions (PPI) underlying the genes altered at both the DNAme and transcriptional highlighted two important functional clusters. One of these was related to the immune response and had the estrogen receptor-encoding *ESR1* gene as its node. The other cluster comprised cardiac electrophysiology-related genes. Intelliectin-1 (*ITLN1*), a component of the innate immune system, was transcriptionally downregulated in HCM and had a hypermethylated site within 1500 bp upstream of the *ITLN1* transcription start site. Estimates of immune infiltration demonstrated a relative decline in immune cell population diversity in HCM. A combination of DNAme and transcriptome profiles may help identify and develop new therapeutic targets for HCM.

## Background

Hypertrophic cardiomyopathy (HCM) is a common hereditary cardiovascular disease with a global incidence of~0.2% [1, 2]. It is a major cause of sudden death and its main clinical manifestation is unexplained left ventricular hypertrophy (≥15 mm). Physiological characteristics of HCM include hypercontraction of the myo fasciculus, impaired diastole, increased sensitivity to calcium ions, and perturbed metabolic signalling [3, 4]. Changes in immune status may also occur during the tissue pathogenesis of HCM [5]. Immunocytes account for 5.3% of the total number of ventricular cells [6]. They maintain cardiac homoeostasis and normal physiological function but also mediate adverse post-injury inflammatory responses, myocardial remodelling, and cardiac electrophysiological abnormalities [7–10]. Cardiac hypertrophy may cause immune dysregulation that negatively impacts cardiac function [7]. HCM patients are at a relatively high risk of sudden death, and heart transplantation is the only efficacious treatment. However, transplantation is often impeded by long wait times because of the shortage of histocompatible donors and the risk of immune rejection. Hence, innovative therapeutic modalities are urgently required to delay the progression of HCM and mitigate the risk of sudden cardiac death associated with it.

DNA methylation (DNAme) is a type of epigenetic modification that affects the entire human genome. It enables the same DNA sequence to be transcribed differently in various cell types. DNAme regulates tissue and organ growth and development. DNAme abnormalities are implicated in the pathogenesis and histopathological changes related to various diseases [11]. CpG hypermethylation in promoter regions downregulates gene transcription. By contrast, CpG hypermethylation in the gene body indicates active gene transcription [12–14]. DNA methyltransferases (DNMTs) and ten-eleven translocation cytosine dioxygenases (TETs) regulate DNAme and demethylation, respectively [15,16].

Aberrant DNAme is involved in the growth and pathological adaptation of cardiomyocytes [17,18]. *DNMT3A* knockdown by CRISPR/Cas9 creates aberrant DNAme in cardiomyocytes which, in turn, modulates the expression of genes associated with altered myocardial contractility, damages the mitochondria, and causes defects in lipid and glucose metabolism [19]. Changes in the DNAme alter the genes encoding potassium channels which play critical roles in cardiac conduction and arrhythmia [20]. Increasing evidence supports the involvement of DNAme in cardiomyopathy, heart failure and transplantation, immune effects and distant effects (e.g., myocardial remodelling and activation of fibrotic processes) [21–30]. In heart failure patients, for instance, satellite (SAT) element hypermethylation is associated with significant upregulation of SAT transcription and may play a role in maintaining chromosomal integrity and myocardial stress response [21,22]. DNAme predicts the risk of complications after transplantation, and FOXP3 promoter hypermethylation predicts immune rejection of heart transplantation [23,24]. In late-stage HCM, some patients may have heart failure. Thus, studies investigating heart failure may detect DNAme alterations occurring in HCM. Nevertheless, dynamic changes in DNAme exist across distinct stages in disease progression [28, 31,32], and the profile for HCM patients prior to heart failure remains unexplored. Furthermore, aberrations of DNAme may regulate innate and adaptive immunity [33–35]. However, little is known about the mechanisms of immune infiltration and aberrant DNAme in HCM myocardium.

We performed a microarray-based genome-wide DNAme analysis paired with transcriptome data to compare HCM against normal left ventricle myocardium. The combination of DNAme screening and transcriptome signals enabled us to investigate HCM mechanisms. It also permitted us to evaluate the effects of the previously hypothesized roles of immune cell function, cardiomyocyte development, and cardiac electrophysiology on HCM. The potential of the immune-related genes identified herein for the treatment of HCM could be explored in subsequent functional studies.

## Methods

### Myocardial specimens

All subjects provided written informed consent prior to participation. All procedures were approved by the Ethics Committee of Beijing Anzhen Hospital (No. 2021155×). Thirty-two myocardial tissue samples were collected from 24 patients with HCM and eight patients with normal myocardia at Beijing Anzhen Hospital. The HCM samples were obtained during septal myectomy and transported to the laboratory in ice-cold cardioplegia solution until cryopreservation (<4 h). HCM diagnosis was defined according to the 2020 AHA/ACC Guideline for the Diagnosis and Treatment of Patients with Hypertrophic Cardiomyopathy [36]. All patients were diagnosed by two experienced clinicians. HCM was confirmed by 2D echocardiography showing unexplained left ventricular hypertrophy with diastolic interventricular septal thickness≥15 mm and septal wall: posterior wall thickness ratio≥1.3 in the absence of any other cardiac or systemic disease causing a similar magnitude of hypertrophy. Normal, healthy myocardial tissues were collected from donor hearts of patients who voluntarily donated their bodies for research. For all donors, clinical examination and medical history displayed no indications of cardiac history nor structural heart disease. All hearts were arrested in situ using ice-cold cardioplegia solution and transported to the lab on wet ice (always<4 hours). The left ventricles (LVs) of hearts were dissected and flash frozen in liquid nitrogen for this study. Tissue DNA was isolated with a QIAmp DNA Mini Kit (Qiagen, Hilden, Germany) and quantified by Qubit fluorometer (Thermo Fisher Scientific, Waltham, MA, USA). Total DNA>0.5 ug was selected and all samples were used for DNAme data collection. Total RNA was isolated from 300 mg tissue with TRIzolTM reagent (Invitrogen, USA) according to the manufacturer’s instruction. RNA concentration was measured using Qubit® RNA Assay Kit in Qubit® 2.0 Fluorometer (Life Technologies, CA, USA). RNA integrity was checked using the RNA Nano 6000 Assay Kit on the Agilent Bioanalyzer 2100 system (Agilent Technologies, CA, USA). The selection criteria were total RNA>0.4 μg, and RNA integrity number (RIN) > 5. Of these, 23 (16 from HCM patients and 7 from normal controls) were qualified for further sequencing.

#### RNA-seq protocol

Poly(A) RNA was purified from 0.4 μg total RNA using Dynabeads Oligo(dT)25–61005 (Thermo Fisher, USA). The poly(A) RNA was fragmented into small pieces using Magnesium RNA Fragmentation Module (NEB, USA). The cleaved RNA fragments were reverse-transcribed into cDNA by SuperScript II Reverse Transcriptase (Invitrogen), which was subsequently used to synthesize U-labelled second-stranded DNAs with E. coli DNA polymerase I (NEB), RNase H (NEB) and dUTP Solution (Thermo Fisher). Size selection was performed with AMPure XP beads (Beckman Coulter, USA). After the heat-labile UDG enzyme (NEB) treatment of the U-labelled second-stranded DNAs, the ligated products were amplified with PCR. The average insert size for the final cDNA library was 300 ± 100 bp. The 2 × 150 bp pair-end sequencing for mRNA were performed on Illumina Hiseq 2500 (Genechem Technology Co. Ltd., Shanghai, China).

#### DNA methylation

DNA was bisulphite-converted with the EZ DNAm kit (Zymo Research) according to the manufacturer’s instructions and then hybridized to the Infinium MethylationEPIC BeadChip (850 K, Illumina). These microarrays were scanned using the Illumina HiScan SQ scanner by Emei Tongde Ltd.

#### Histology

HCM and control LV tissue samples were successively perfused by saline and 4% paraformaldehyde. LV tissues were fixed for 24 h (4%paraformaldehyde), transferred to ethanol (70%) for subsequent dehydration and paraffin embedding. LV samples were continuously cut into sections, and stained with Masson’s trichrome and Hematoxylin‑eosin (HE) staining by following the protocols.

### Variant calling and annotation

Read mapping and variant calling were performed according to the Broad Institute GATK [37] best practices workflow for SNP and Indel calling on RNA-seq data (https://software.broadinstitute.org/gatk/documentation/article.php?id=3891). Briefly, paired-end reads were mapped onto the human reference genome (b37) by the STAR two-pass alignment method [38]. Picardtools (http://broadinstitute.github.io/picard/) was used to add and sort read groups, mark duplicates, and create an index. The SplitNCigarReads function (https://gatk.broadinstitute.org/hc/en-us/articles/9570487998491-SplitNCigarReads) was used to split the reads into exon segments. BaseRecalibrator (https://gatk.broadinstitute.org/hc/en-us/articles/360036898312-BaseRecalibrator) and ApplyBQSR (https://gatk.broadinstitute.org/hc/en-us/articles/360037055712-ApplyBQSR) were used to correct for systematic bias affecting the assignment of base quality scores by the sequencer. Variant calling was performed with HaplotypeCaller (https://gatk.broadinstitute.org/hc/en-us/articles/360037225632-HaplotypeCaller) and annotated with wANNOVAR (https://wannovar.wglab.org) [39]. Variants in the exons of the HCM pathogenic genes (*MYBPC3*, *MYH7*, *MYL2*, *MYL3*, *TNNI3*, *TNNT2*, *TPM1*, and *ACTC1*, and other 3769 HCM-related genes listed in Genecards) were screened from the annotation results [2].

### DNAme data QC and differentially methylated probe (DMP) analysis

The bisulphite treatment in the EZ DNA Methylation-Gold Kit was used to convert the DNA samples according to the manufacturer’s instructions (Zymo Research, Irvine, CA, USA). DNAme was detected with an Infinium Human Methylation EPIC BeadChip (850k) Microarray (Illumina, San Diego, CA, USA). Raw fluorescence data for the DNAme were stored in IDAT files. The ‘ChAMP’ package (https://bioconductor.org/packages/release/bioc/html/ChAMP.html) in R (R Core Team, Vienna, Austria) was used for QC and data analysis. Raw data were loaded with the ‘champ.load’ function and transformed into β-values. The latter represent the ratios of the fluorescence intensities of methylated probes to those of unmethylated probes and range from 0 to 1. The β-value increases with the degree of methylation. This function was also used for initial probe screening and QC. Probes meeting any of the following criteria were removed: (1) *p* > 0.01; (2) < 3 beads in≥5% of all samples; (3) non-CpG; (4) multihits; (5) underlying SNPs; and (6) location on X or Y chromosome. The filtered probe set was used in the subsequent analyses. Beta mixture quantile expansion (BMIQ) normalization and singular value decomposition (SVD) were used to eliminate batch effects caused by differences in experimental time points. A principal component analysis (PCA) was performed using the ‘prcomp’ function in the ‘stats’ package (https://statisticsglobe.com/stats-r-package) of R. PCA reduced the dimensionality of the high-dimensional methylation site matrix/gene expression matrix into a form with only a few principal components. This small number of principal components can then effectively represent the variation at all methylation sites/gene expression, and therefore describes the differences between samples.

The ‘Bumphunter’ method (https://bioconductor.org/packages/release/bioc/html/bumphunter.html) was used to screen DMPs. The △β-value is the arithmetic difference between the mean β-value of the HCM group and that of the normal control for a single probe. Correction of *P* values (adjusted P) for multiple comparisons was performed by using the Benjamini-Hochberg procedure. The DMP thresholds were adjusted P < 0.05 and log|FC| > 0.1. The latter term is equivalent to the effect of |△β-value| > 0.1. HCM_hyper_ DMPs had logFC>0.1 whereas HCM_hypo_ DMPs had logFC<0.1. DMP gene annotations were performed using the default annotation of the ‘ChAMP’ package in R. The reference genome was hg19 (https://www.ncbi.nlm.nih.gov/assembly/GCF_000001405.13/).

An in-house Python script was used to evaluate probe overrepresentation in each chromosome. To determine DMP overrepresentation in a chromosome in all DMPs (DMPSet), it was necessary to determine the *a priori* distribution of the number of probes (N) from a chromosome in a random probe set (ProbeSet^R^) equal in size (M) to the DMPSet. To this end, M probes were sampled 10,000 times with replacements from all probes in the array, and the cumulative probability of observing *N* > M within each chromosome in a ProbeSet^R^ was determined. For HCM_hyper_ DMPs and HCM_hypo_ DMPs, 2,202 and 1,864 probes, respectively, were randomly sampled 10,000 times from 732,724 total probes in the array. The significance of the overrepresented chromosome was the sum of the probabilities (P-values) of finding *N* > M probes from this chromosome in the DMPSet. *P* value<0.05 indicated that the number of probes was significantly increased on the chromosome.

### RNA-seq data QC and differential expression analysis

RNA was extracted and purified from 16 HCM and seven normal samples. Sequencing libraries with insert fragment length = 380 bp were sequenced on the Illumina HiSeq X ten platform (paired-end; 2 × 150 bp) and generated 6Gb raw data/sample. Raw reads were filtered with Cutadapt (https://pypi.org/project/cutadapt/) to remove 3’ adapters with≥10 bp overlap (AGATCGGAAG) while allowing for a 20% base error rate. FastQC (https://www.bioinformatics.babraham.ac.uk/projects/fastqc/) was used to filter the reads using the default QC parameters. HISAT2 (http://daehwankimlab.github.io/hisat2/) was used to align the filtered reads to the GRCh38 reference genome (https://www.ncbi.nlm.nih.gov/data-hub/genome/GCF_000001405.40/). The ‘Union’ scheme in HTSeq (https://pypi.org/project/HTSeq/) was used for counting. Raw read counts were analysed with DESeq2 (https://bioconductor.org/packages/release/bioc/html/DESeq2.html). Those with adjusted *P* < 0.05 and log_2_|FC| > 1.0 were considered differentially expressed genes (DEGs). PCA was performed using the built-in function in DESeq2.

### Gene set enrichment analysis (GSEA) and gene ontology (GO) and Kyoto Encyclopedia of Genes and Genomes (KEGG) enrichment analyses

GSEA and GO and KEGG enrichment analyses were performed using the ‘clusterProfiler’ package (https://bioconductor.org/packages/release/bioc/html/clusterProfiler.html) in R [40]. All DMPs were annotated with hg19 to obtain the DMP genes. The GO (adjusted P < 0.05) and KEGG(*P* value<0.05) enrichment analyses were performed on all DMP genes and DEGs. The significantly enriched GO items were grouped in either dataset into the functional network within ClueGO v. 2.5.9 (https://apps.cytoscape.org/apps/cluego) in Cytoscape v. 3.9.1 (https://github.com/cytoscape/cytoscape/releases). The default parameters were edge-weighted, force-directed, and BioLayout (http://biolayout.org/download.html) for CluePedia (https://apps.cytoscape.org/apps/cluepedia). Cytoscape was used to construct a network from the overlapping pathways in the results of the GO enrichment analyses of the DMP genes and the DEGs. The κ (kappa) score threshold was 0.3 and the sharing group percentage was 30.0%. GSEA was applied only to DEGs with adjusted P < 0.05.

### Protein-protein interaction (PPI) network analysis and pathogenic gene screening for overlapping genes of DMP genes and DEGs

A PPI network of overlapping genes was constructed based on the STRING (https://string-db.org/), an online protein network database [41]. Genes with moderate confidence interaction scores (>0.4) were selected and isolated node genes were removed. The remaining genes were clustered into subnetworks by the Markov clustering (MCL) method. The inflation parameter was 1.5. All other parameters used in this analysis were set by default. The immune-related gene dataset that was confirmed in earlier studies to be implicated in the natural immune process was downloaded from the IMMPORT database [42] (https://www.immport.org/shared/). The GeneCards (https://www.genecards.org/) database was used to screen and obtain pathogenic HCM genes. The keyword was ‘hypertrophic cardiomyopathy’ [43].

### xCell immune infiltration analysis

The xCell analysis uses a deconvolution algorithm and integrates the advantages of GSEA. The xCell calculated single-sample gene set enrichment analysis (ssGSEA) scores for 489 gene signatures. Gain compensation was then corrected and new scores were used to assess the relative abundance of 64 cell types in each tissue sample. Cell types included multiple adaptive and innate immune, haematopoietic progenitor, epithelial, and extracellular stromal cells as well as 48 TME-associated cells. Gene expression values (fragments per kilobase per million mapped reads or FPKM) from the bulk RNA-seq data were uploaded to http://xCell.ucsf.edu/ [44] to obtain 64 cell scores per sample. Immune cells with *P* value<0.05 *(t*-test) were selected for display.

### Public HCM and normal myocardium RNA-seq data

The public GSE130036 dataset (https://www.ncbi.nlm.nih.gov/geo/query/acc.cgi?acc=GSE130036) contains raw RNA-seq data for 28 HCM and nine normal myocardial samples. Trimmomatic (https://github.com/usadellab/Trimmomatic) was used to remove the adapter, and FastQC (https://www.bioinformatics.babraham.ac.uk/projects/fastqc/) with its default parameters was used for quality control. HISAT2 (http://daehwankimlab.github.io/hisat2/) compared the filtered reads against the GRCh38 reference genome. The ‘Union’ scheme in HTSeq (https://pypi.org/project/HTSeq/) was used for counting.

### Data availability

The raw DNAme data (No. PRJCA009134; https://ngdc.cncb.ac.cn/omix/preview/ocAxwKJt) and the raw RNA-seq data (No. PRJCA009145; https://ngdc.cncb.ac.cn/gsa-human/s/mRwWy2L3) reported herein were deposited to the Genome Sequence Archive [45] of the China National Center for Bioinformation/Beijing Institute of Genomics of the Chinese Academy of Sciences and is publicly accessible.

## Results

### Clinical characteristics of study patients and donors

**Figure 1.**
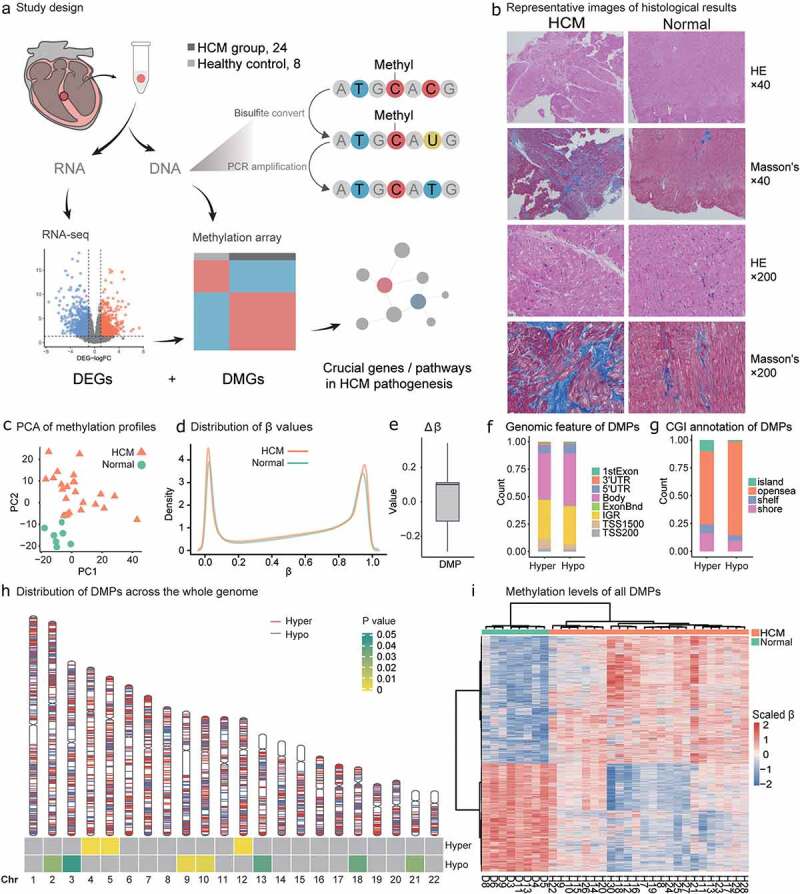
**Study design and HCM methylation landscape**. (a) HCM samples were obtained during septal myectomy. Twenty-four HCM and eight normal control samples were used for DNAme data collection. Sixteen HCM and seven normal control samples were used for RNA-seq data collection. Differential methylation, differentially expressed gene, and combinatorial analyses were performed to identify key genes and pathways in HCM pathogenesis. (b) Representative images of histological results. HE staining and Masson’s staining results of LV from HCM patients and normal control. Scale bar, 50 μm. (c) Principal component analysis (PCA) of DNAme in 32 samples revealed overall differences between HCM and normal samples. (d) β-value density distribution. HCM myocardium harboured hypomethylated and hypermethylated DNA. (e) △β-value distribution and range of DMPs; −0.29 to−0.1 for HCM_hypo_ DMPs and 0.1 to 0.34 for HCM_hyper_ DMPs. (f) DMP distributions in gene models. (g) DMP distributions in CpG islands (CGIs) (h) DMP distributions across autosomes. Red and blue lines on each chromosome indicate hypermethylation and hypomethylation sites, respectively. Heatmap at the bottom shows the significance of HCM_hyper_ DMP and HCM_hypo_ DMP enrichment for each chromosome. Scaled colour bar = p-value. (i) DMP methylation levels in 32 samples. Scaled colour bar = β-value.

Thirty-two cardiac ventricular septal tissue samples were collected from 24 HCM patients and eight donors (Methods; Table 1). DNAme profiles were determined for all samples, and paired RNA-seq analyses were performed on 23 samples (Figure 1a). The HCM group consisted of 14 males and 10 females with average age 56 ± 13 y and maximum left ventricular wall thickness (LVST) = 19.3 ± 4.4 cm. There was no significant difference between the sexes in terms of their maximum LVST (*P* = 0.6854; *t*-test). HE staining results indicated myocyte hypertrophy, myocyte disarray, nuclear hyperchromatism, and vacuolar degeneration in HCM patients compared with normal controls in 40× and 200× views. Interstitial fibrosis and replacement fibrosis were detected in Masson-stained samples of HCM patients(Figure 1b). The HCM patients harboured different variants in the exons of their pathogenic HCM genes *MYBPC3*, *MYH7*, *MYL2*, *MYL3*, *TNNI3*, *TNNT2*, *TPM1*, and *ACTC1* [2]. No major pathogenic variants were responsible for patient stratification (Methods; Supplementary Table S1). We did not find significant differences in either methylome or transcriptome in terms of gender (male, or female), BMI (>24 or<24) and diabetes (with or without).

**Table 1.** Clinical characteristics of HCM patients.

Sample ID	Sex	Age	BMI(kg/m^2^)	Hypertension	Diabetes	Maxi LVST(mm)	LVEDD(mm)	LVESD(mm)	LVEF(%)	LVOTG(mmHg)	SAM
H7	F	70	19.5	NO	YES	15	33	NA	60	142	+
H8	M	46	25.8	NO	NO	20	45	29	66	56	+
H9	F	68	27.8	NO	NO	19	41	27	64	85	+
H10	M	58	22.6	NO	NO	18	43	25	66	59	+
H11	F	69	19.6	NO	YES	20	45	28	68	156	+
H12	M	74	27.3	YES	NO	15	45	30	60	55	+
H13	M	55	27.8	NO	NO	19	39	22	73	65	+
H14	M	47	27.4	NO	NO	17	50	35	56	49	+
H15	M	43	27.4	NO	NO	23	43	26	70	113	+
H16	F	61	26.0	NO	NA	29	35	21	74	90	+
H17	M	44	NA	NO	NO	25	42	26	67	84	+
H18	F	65	NA	NO	NA	18	45	29	68	135	NA
H19	F	44	27.8	NO	NO	19	42	25	70	94	+
H20	F	70	20.4	NO	YES	15	47	27	73	119	+
H21	M	13	NA	NO	NO	30	35	20	72	51	+
H22	M	49	24.9	NO	NO	14	42	25	73	101	+
H23	M	61	24.5	NO	NO	17	45	29	65	55	+
H24	M	61	24.5	NO	NO	17	45	29	65	55	+
H25	M	49	33.5	NO	NO	21	41	22	75	68	+
H26	F	70	NA	NO	NO	12	46	31	60	97	+
H27	M	64	27.4	NO	YES	17	53	36	43	57	+
H28	F	54	24.8	NO	NO	18	53	30	71	89	+
H29	F	57	29.3	NO	NA	23	46	30	62	42	+
H30	M	46	27.7	YES	NO	21	44	30	62	90	+

**Maxi LVWT**: maximum left ventricular wall thickness; **LVEDD**: Left ventricular end diastolic diameter; **LVESD**: Left ventricular end systolic diameter; **LVEF**: left ventricular ejection fraction; **LVOTG**: left ventricular outflow track gradient; **SAM**: systolic anterior movement; **NA**: not available.;’**+**:’positive.

### DMPs: methylation level and chromosome distribution

After quality control, 732,724 CpG probes were obtained and used to cluster 32 samples into two groups consistent with disease grouping (PCA; Methods; Figure 1c). The HCM myocardium presented with both hypomethylation and hypermethylation changes and exhibited elevated double β-value distribution peaks (Methods; Figure 1d). There were 4,066 DMPs (adjusted *P* < 0.05; log|FC| > 0.1; Methods; Supplementary Table S2) of which 54% were hypermethylated (HCM_hyper_ DMPs; Methods). Fluctuations in the hypermethylation and hypomethylation alterations were indicated by the △β-value ranges (Methods; Figure 1e). These were −0.29 to −0.1 for the HCM_hypo_ DMPs and 0.1 to 0.34 for the HCM_hyper_ DMPs.

The DMPs were unevenly distributed across the chromosomes (Genomic annotation; Methods). The HCM_hyper_ DMPs were enriched on chromosomes 4,5, and 12 while the HCM_hypo_ DMPs were located on chromosomes 2, 3, 9, 10, 13, 18, and 21 (*P* value<0.05, Figure 1h). In terms of genomic distribution (Figure 1f,g), 48% and 42% of the HCM_hypo_ DMPs and HCM_hyper_ DMPs, respectively, were located within the gene body while 84% and 66% of HCM_hypo_ DMPs and HCM_hyper_ DMPs, respectively, were located within the opensea. Overall, the methylation levels of the 4,066 DMPs significantly differed and could effectively discriminate the HCM myocardium from the normal tissues (Figure 1i).

### Hypermethylation and hypomethylation disturb crucial cardiac cell functions in HCM pathogenesis and are associated with corresponding changes in transcriptional activity

DMPs were annotated for 1,927 genes (DMP genes; Genomic annotation; Methods). Hyper-DMPs and hypo-DMPs influenced distinct cellular functions. The HCM_hyper_ DMP genes were associated with cardiac growth and development processes such as heart morphogenesis (Gene ontology enrichment; Methods; adjusted *P* = 1.02 × 10^−5^), cardiac chamber morphogenesis (adjusted *P* = 1.22 × 10^−5^), cardiac chamber development (adjusted *P* = 1.22 × 10^−5^), and outflow tract morphogenesis (adjusted *P* = 1.83 × 10^−5^, Supplementary Figure S1A). However, the HCM_hypo_ DMP genes were correlated with muscle structure-related processes including actin binding (adjusted *P* = 4.19 × 10^−5^), myofibrils (adjusted *P* = 3.95 × 10^−4^), sarcomeres (adjusted *P* = 7.11 × 10^−4^), contractile fibres (adjusted *P* = 7.34 × 10^−4^), contractile actin filament bundles (adjusted *P* = 7.34 × 10^−4^), and stress fibres (adjusted *P* = 7.34 × 10^−4^, Supplementary Figure S1B).

**Figure 2.**
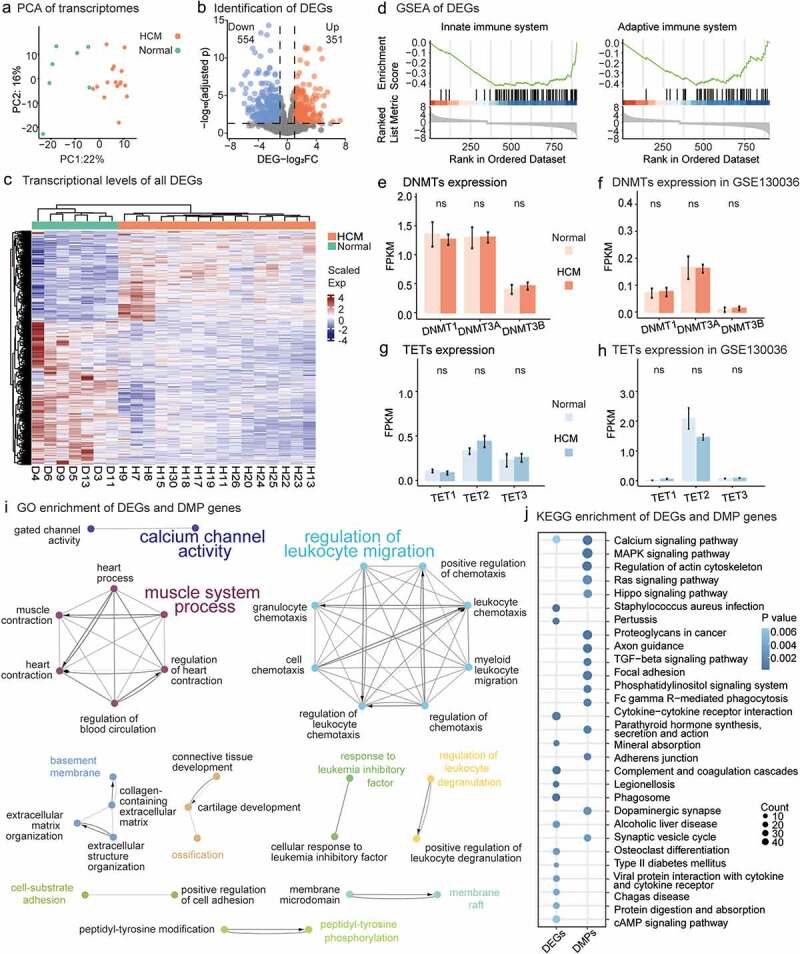
Differentially expressed genes (DEGs) in HCM and DEGs and DMP gene enrichment analyses. (a) PCA of gene expression profiles for all 32 samples. (b) DEGs with high/low expression. Compared with healthy samples, HCM samples had 554 downregulated and 351 upregulated genes. (c) DMP transcription levels in HCM and healthy groups. Scaled colour bar = FPKM values. (d) GSEA of DEGs related to the ‘innate immune system’ and ‘adaptive immune system.’ DMMT transcriptional levels. No significant intergroup differences were observed in our data (e) or GSE130036 public dataset (f). No transcriptional changes were observed for TETs in our data (g) or GSE130036 public dataset (h). (i) Functionally grouped networks of DEG and DMP gene-enriched GO items in enrichment analysis of both gene sets. (j) Fifteen most significant KEGG-enriched pathways of DEGs and DMP genes. Circle size is proportional to the number of genes in the pathway. Color represents P-value.

A paired RNA-seq analysis of the 23 samples was performed to identify gene expression alterations related to aberrant DNAmes (Supplementary Table 3; PCA, Methods; Figure 2a). We identified 905 DEGs in the HCM samples (adjusted *P* < 0.05, log_2_|FC| > 1.0, Methods; Supplementary Table 4; Figure 2b,c). The DEGs were significantly enriched in the pathways related to the innate immune system (GSEA; enrichment score = −0.43; adjusted *P* = 1.24 × 10^−3^; Figure 2d, left) and the adaptive immune system (GSEA enrichment score = −0.43; adjusted *P* = 1.79 × 10^−2^; Figure 2d, right). Their transcriptional activity was decreased in the HCM myocardium.

A GO enrichment of the DEGs and DMP genes revealed pathways with extensive DNAme and gene expression alterations that were functionally grouped into various networks (Methods; Figure 2i). Two major immune subnetworks included most of the GO items. One was characterized by ‘regulation of leukocyte migration’ while the other was characterized by ‘muscle system process.’ Calcium signalling was the only significantly enriched KEGG pathway for both the DMP genes (*P* value = 2.18 × 10^−4^) and the DEGs (*P* value = 6.04 × 10^−3^) (Figure 2j).

The transcriptional activity of the DNMTs and TETs did not explain the observed changes in HCM DNAme. There were no significant differences in transcription between HCM myocardium and normal tissues for any DNMT family member genes (*DNMT1*, *DNMT3A*, and *DNMT3B*; *P* value>0.05; *t*-test; Figure 2e) or TET-related genes (*TET1*, *TET2*, and *TET3*; *P* value>0.05; *t*-test; Figure 2g). A public HCM dataset validated our observations (*P* value>0.05; *t*-test; Figure 2f,h). Of the methyl-CpG-binding domain (MBD) proteins or epigenome readers [46], *EGR1* was downregulated and *BAZ2B* was upregulated, but there were no DNAme alterations (Supplementary Table S4). Changes in DNAme were detected in *MBD4*, *ZBTB38*, *KLF4*, *WT1*, and *UHRF2* but without any obvious alteration of transcription (Supplementary Table S2). Compared with previous observations in human cardiomyocytes (hCMs) [47], DMP genes influenced all three constructed function groups: muscle contraction, cardiac transcriptional regulation, and heart development (Supplementary Table S5).

### The nodes of the PPI network of DEGs harbouring DMPs underscored the roles of immune regulation, cardiac development, and electrophysiology in HCM pathogenesis

**Figure 3.**
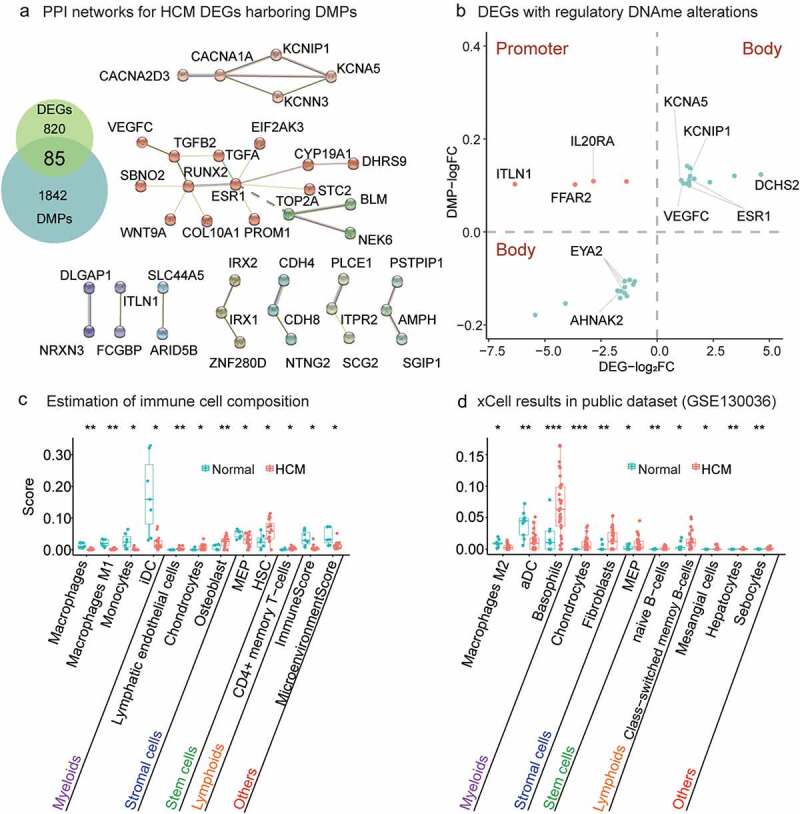
**Relationship between gene methylation and transcription and results of xCell analysis**. (a) PPI networks of HCM DEGs harbouring DMPs. PPIs network of 79 DEGs affected by DNAme alterations consisted of two major clusters. A larger cluster harboured genes related to tissue and organ growth and development and was associated with genes regulating immune functions such as *ESR1*, *STC2*, *TGFA*, *TGFB2*, and *VEGFC*. (b) Twenty-five DEGs with regulatory DNAme alterations. CpG hypermethylation in gene promoters downregulates gene transcription (orange dots). CpG hypermethylation/hypomethylation in the gene body indicates/downregulates active gene transcription (green dots). Estimation of immune cell composition. Immune cells with distinct enhancement scores in HCM myocardium compared with normal tissue in xCell analysis are shown with their significance levels (*P value<0.05, **P value<0.01, ***P value<0.001) for our data (c) and GSE130036 public dataset (d). Immune cells arranged in order ‘Myeloids,’ ‘Stromal cells,’ ‘Stem cells,’ ‘Lymphoids,’ and ‘Others’.

To identify DEGs caused by DNAme alterations, we extracted 85 genes common to both the DEGs and the DMP genes (Supplementary Table 6; Figure 3a, left). Twenty-four percent (20/85) of them were HCM genes in GeneCards (Supplementary Table 7). *TTN-AS1* was the most relevant gene (score = 169) to HCM. Hence, these disease-causing genes may also have DNAme alterations and contribute to HCM occurrence. Nine of the 85 common genes, namely, *ESR1*, *GDF6*, *IL20RA*, *PMP2*, *SCG2*, *STC2*, *TGFA*, *TGFB2*, and *VEGFC*, are associated with cytokines, cytokine receptors, the TGFb family, interleukin receptors, and antimicrobials (IMMPORT database; Supplementary Table 8). PPI networks were resolved for 79 DEGs with aberrant DNAme (Methods; Figure 3a, right). Genes associated with disease causality, namely, *ESR1*, *RUNX2*, and *CACNA1A*, were crucial nodes with edges>3. *ESR1* and *RUNX2* belonged to the larger of the two clusters associated with tissue and organ growth and development. *CACNA1A* was the central node of the cluster involved in cardiac electrophysiology.

Twenty-five DEGs could be explained by the regulatory roles of DNAme alterations (Methods; Figure 3b). According to the enriched KEGG and GO pathways, the DEGs were involved in biological processes related to immune response, myocardial growth and development, cardiac conduction, and electrophysiology. The immune response-related genes included *ESR1* [48, 49], *ITLN1* [50, 51], *AHNAK2* [52, 53], *FFAR2* [54–56], *IL20RA* [57, 58], and *VEGFC* [59, 60]. The myocardial growth and development-related genes included *ESR1* [61, 62], *ITLN1* [63, 64], *EYA2* [65, 66], *VEGFC* [67], and *DCHS2* [68]. The myocardial conduction and electrophysiology-related genes included *ESR1* [69], *KCNIP1* [70,71], *KCNA5* [72, 73], and *AHNAK2* [74].

The HCM_hyper_ DMPs in the gene body indicated transcriptional upregulation of *ESR1* and *VEGFC* while those in the promoter might explain *IL20RA* downregulation (Table 2). *DCHS2* was the most significantly upregulated gene (log2FC = 4.6; adjusted *P* = 2.17 × 10^−3^; Table 2) and it encodes a calcium-dependent cell-adhesion protein. *ITLN1* was the most significantly downregulated gene (log2FC = − 6.3, adjusted *P* = 3.15 × 10^−7^; Table 2).

**Table 2.** DNA methylation and transcriptional changes of important genes.

Gene symbol	Function	Gene transcription information			DNA methylation information				
		log_2_FoldChange	P value	P adj	DMP-ID	logFC	P value	Adj P Val	feature
*ESR1*	immune response; myocardial growth and development; myocardial conduction system and electrophysiology	1.43	3.30E–05	1.88E–03	cg26581065	0.11	3.33E–05	2.10E–03	Body
					cg02285263	0.10	3.68E–03	3.47E–02	Body
*ITLN1*	immune response; myocardial growth and development	−6.33	5.91E–10	3.15E–07	cg08356693	0.10	3.70E–06	5.99E–04	TSS1500
*AHNAK2*	immune response; myocardial conduction system and electrophysiology	−1.62	1.26E–04	4.75E–03	cg08508153	−0.13	1.40E–05	1.28E–03	Body
*VEGFC*	immune response; myocardial growth and development	1.17	1.26E–04	4.73E–03	cg25560196	0.10	5.84E–05	2.91E–03	Body
*FFAR2*	immune response	−3.65	3.03E–05	1.77E–03	cg02361306	0.10	3.95E–03	3.63E–02	TSS200
*IL20RA*	immune response	−2.84	3.16E–08	8.53E–06	cg08823985	0.11	6.34E–10	4.38E–06	TSS1500
*DCHS2*	myocardial growth and development	4.62	4.12E–05	2.17E–03	cg07436354	0.12	1.17E–04	4.33E–03	Body
*EYA2*	myocardial growth and development	−1.44	8.57E–04	1.82E–02	cg02619733	−0.12	2.31E–06	4.58E–04	Body
					cg26273742	−0.11	4.00E–04	8.99E–03	Body
					cg17485169	−0.13	2.56E–06	4.88E–04	Body
*KCNA5*	myocardial conduction system and electrophysiology	1.07	1.60E–03	2.77E–02	cg16399511	0.11	1.41E–05	1.28E–03	1stExon
*KCNIP1*	myocardial conduction system and electrophysiology	1.47	9.59E–04	1.95E–02	cg16300021	0.12	1.05E–06	2.88E–04	Body

### Dysregulated immune responses were observed in cardiac hypertrophy and remodelling

The heart consists of heterogeneous cell subtypes intensively involved in cardiac hypertrophy and remodelling [6,75]. We estimated the immunocyte composition (macrophages, T cells, and dendritic cells (DCs)) of the HCM myocardium using the novel gene signature-based xCell method (xCell; Methods). The ImmuneScore was significantly lower for the ventricle tissues of the HCM group than it was for those of the normal group. Macrophages comprise a wide range of functionally heterogeneous phenotypes. Understanding the roles of various macrophage phenotypes during cardiac remodelling could help develop a promising therapeutic strategy [7]. In the present study, we found that the total macrophages (*P* value<0.01; *t*-test) and M1 macrophages (*P* value<0.01; *t*-test) were significantly decreased in HCM patients (Figure 3c). Macrophages respond to different environmental signals and activate various polarization programs [76, 77]. Similarly, monocytes (*P* value<0.05; *t*-test) and induced dendritic cells (iDCs; *P* value<0.05; *t*-test) were significantly decreased in HCM patients. Relative to the public dataset, our results showed that the active dendritic cells (aDCs) (*P* value<0.01; *t*-test) and M2 macrophages (*P* value<0.05; *t*-test) were significantly decreased in HCM myocardial tissue (Methods; Figure 3d). The preceding results reflect end-stage HCM which might differ from early disease onset.

The stromal cells were significantly increased in the public datasets (GSE130036) as well as our own compared with the normal group (Figure 3c,d). The foregoing findings indicate that nonmyocytes in general and immune cells, in particular, were dysregulated in HCM patients. The immune response and regeneration capacity may be significantly impaired in HCM patients as their immune, stromal, and stem cells are all dysregulated. Nevertheless, the mechanisms of these dysregulations remain to be elucidated and merit further investigation.

## Discussion

DNAme alterations have been detected in various diseases and are promising as diagnostic and prognostic biomarkers [78]. As an inherited disease, abnormal myocardial function due to pathogenic genomic alterations is anticipated to be an important aetiological factor of HCM, and we believe that altered DNA methylation may be a secondary alteration in myocardial function compensation. Recent progress has shown methylation changes may result from altered transcription activity in response to a stimulus in cardiomyocytes [79]. The authors found that the acute hypoxia stress response continuously activated specific gene expression patterns and resulted in DNAme changes in regulatory regions of the corresponding genes. Therefore, transcriptional and DNAme changes help elucidate these molecular mechanisms of HCM pathogenesis. However, it requires considerable effort to determine DNAme profiles for human cardiac tissues. Animal models of heart failure and tissue samples from heart failure patients showed DNAme alterations in targeted genomic regions [27–29], here we further provided DNAme profiles from HCM patients in surgery prior to heart failure to explore changes in a relatively earlier stage in disease progression. Unlike tumour cells, HCM cardiomyocytes retain their basic morphology and functions. As transcriptional changes in methylation-related enzymes do not occur in HCM myocardium, there may be fewer changes in HCM DNAme than in the tumour genome. Abnormal gene expression in tumours may explain the extensive epigenetic reprogramming characteristic of genome-wide DNAme alterations. Future research should endeavour to investigate whether changes in DNMT and TET protein abundance occur in human HCM myocardium and identify their contributions to alterations in HCM DNAme. Besides, methylation quantitative trait loci (meQTL)/Expression quantitative trait loci (eQTL) analysis was used to correlate genetic variant loci with DNAme levels at specific CpG loci/expression of specific genes to explain genetic variation in the disease [80, 81]. With accumulation of this type of data for HCM, meQTL/eQTL analysis will provide a better explanation of genetic variation in familial HCM.

Myocardial hypercontraction, dysregulation of calcium homoeostasis, and metabolic signalling disorders are pathological manifestations of HCM [4, 82–84]. To the best of our knowledge, the present study provided DNAme profiles for HCM tissues with paired transcriptome datasets and determine the alterations in DNAme underlying changes in gene expression. DNAme profiles facilitate the exploration of the connections underlying myocardial remodelling and cardiac electrophysiological abnormalities during pathogenesis. They may also disclose candidate targets for the development of novel therapeutic strategies for HCM.

We observed decreases in innate and adaptive immune activity as well as mononuclear macrophage system-related components in the HCM myocardium. These changes are closely related to cardiac electrophysiological abnormalities and myocardial remodelling. Macrophage depletion leads to abnormalities in cardiac electrical signalling and especially atrioventricular block and predisposes the heart to progressive cardiomyopathy, reduced cardiac output, diastolic dysfunction, and impaired haemodynamics [10, 85]. The most significant change in HCM myocardium was a reduction in iDC content. These antigen-presenting cells have a strong migratory capacity, and various types of iDCs reside in the myocardium [86]. In dilated cardiomyopathy, apoptosis and insufficient angiogenesis decrease DC diversity and increase the number of mature DCs [87]. This mechanism may explain the reductions in iDCs observed in HCM myocardium. In comparison, DEGs in hCMs were enriched only in pathways associated with cell lineage and tissue differences rather than immune-related ones [47]. Immune cell population changes in HCM are likely to result from myocardial remodelling or myocardial injury, but further efforts are required to dissect their impacts on cardiomyocytes. Future integration of high-resolution single-cell methylation sequencing and single-cell transcriptome sequencing may reveal methylation alterations in immune cell and cardiomyocyte interactions.

We found that ESR1 encodes a cytokine receptor also as a transcription factor that has motifs for estrogen binding, DNA binding, and transcription activation. It had the most edges in the HCM PPI network. The two HCM_hyper_ DMPs within its gene body may explain its upregulated transcription level. Overexpression of the *ESR1* protein product ERα might be a protective factor in disease progression, prevent cardiac hypertrophy [61, 62, 88], reduce arrhythmias [69], improve vascular endothelial function [89], and modulate innate immune signalling in DCs and macrophages [49]. There were no differences between the sexes in terms of their myocardial *ESR1* expression levels [90–92]. Nevertheless, males have relatively higher incidence and significantly lower average age at HCM diagnosis than females [93–96]. The hormone receptor *ESR1* might help maintain basic physiological functions in lesions and slow disease progression. Future investigations into the therapeutic mechanism of *ESR1* might be warranted.

*ITLN1* underwent the most significant transcriptional changes and corresponding DNAme alterations in all HCM samples. In HCM myocardium, *ITLN1* has a hypermethylated site within 1500 bp upstream of the transcriptional start site, which may lead to its transcriptional down-regulation. *ITLN1* is a component of the innate immune system. It is an anti-inflammatory adipocytokine present in epicardial adipose tissue and it has cardioprotective efficacy [50]. A recent study demonstrated altered transcriptional activity in failing ventricles [97]. Our HCM patients displayed significantly downregulated *ITLN1* and, therefore, limited macrophage differentiation into the anti-inflammatory M2 phenotype [51]. We also observed decreased macrophage composition in the transcriptome dissection for our HCM samples. Hence, *ITLN1* downregulation is a key factor in this phenomenon. Although the present work characterized HCM at the DNA and RNA levels, these bulk tissue data can not identify changes specific to a cell population, and future efforts are required to determine the DNAme alterations in cardiocytes and validate the composition changes of immune cells in HCM myocardium.

In the present study, we profiled the changes in DNAme in HCM myocardium and evaluated their corresponding impact by jointly analysing DNAme and the transcriptome. We built upon traditional pathological alterations in HCM and identified possible links among immune dysregulation, cardiac electrophysiological abnormalities, and myocardial remodelling. Immune-related genes that also regulate DNAme might help identify and develop novel therapeutic strategies for HCM.

## Supplementary Material

Supplemental Material

## Data Availability

The datasets supporting the conclusions of this article are available in the Genome Sequence Archive [45]. The raw DNAme data are hyperlinked to the dataset at https://ngdc.cncb.ac.cn/omix/preview/ocAxwKJt while the raw RNA-seq data are hyperlinked to the dataset at https://ngdc.cncb.ac.cn/gsa-human/s/mRwWy2L3.
