# Supplementary material for: DNA methylome and transcriptome profiling reveal key electrophysiology and immune dysregulation in hypertrophic cardiomyopathy
Source: Epigenetics. 2023 Apr 2;18(1):2195307. doi: 10.1080/15592294.2023.2195307 (PMC10072074; doi:10.1080/15592294.2023.2195307)
Supplement: Supplemental Material [file KEPI_A_2195307_SM9987.zip › Supplementary files/Supplementary Figure legends.docx]

**Supplementary Files**

**Supplementary Figure 1. GO enrichment analysis of DMP genes.** (A) GO enrichment analysis results of HCM_hyper_ DMP genes. (B) GO enrichment analysis results of HCM_hypo_ DMP genes. BP, biological process; MF, molecular function; CC, cellular component.

**Supplementary Table 1. Variants of HCM-causing genes.**

**Supplementary Table 2. DMPs between HCM patients and healthy individuals.**

**Supplementary Table 3. RNA sequencing quality.**

**Supplementary Table 4. DEGs between HCM patients and healthy individuals.**

**Supplementary Table 5. DMP genes of muscle contraction, cardiac transcriptional regulation, and heart development.**

**Supplementary Table 6. Overlapping DMP genes and DEGs.**

**Supplementary Table 7. Genes regulating HCM pathogenicity.**

**Supplementary Table 8. Overlapping DEGs and DMP and ImmPort dataset genes.**
