# Supplementary figures and images for: DNA methylome and transcriptome profiling reveal key electrophysiology and immune dysregulation in hypertrophic cardiomyopathy
Source: Epigenetics. 2023 Apr 2;18(1):2195307. doi: 10.1080/15592294.2023.2195307 (PMC10072074; doi:10.1080/15592294.2023.2195307)

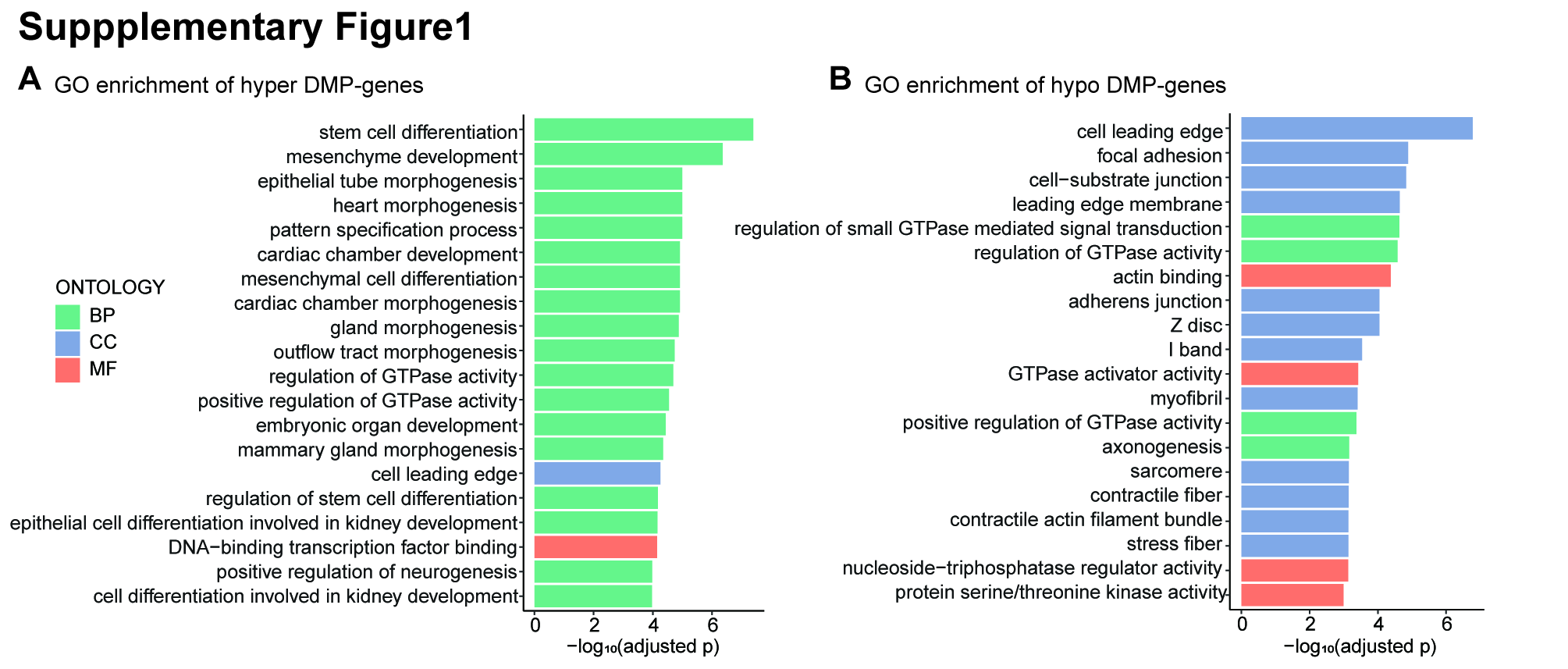

Supplement: Supplemental Material [file KEPI_A_2195307_SM9987.zip › Supplementary files/suppplementary Figure1_Revised.tif]
